# Sudden neonatal death in individuals with medium-chain acyl-coenzyme A dehydrogenase deficiency: limit of newborn screening

**DOI:** 10.1007/s00431-022-04421-y

**Published:** 2022-03-16

**Authors:** Ulrike Mütze, Uta Nennstiel, Birgit Odenwald, Claudia Haase, Uta Ceglarek, Nils Janzen, Sven F. Garbade, Georg F. Hoffmann, Stefan Kölker, Dorothea Haas

**Affiliations:** 1 Division of Child Neurology and Metabolic Medicine, Department of General Pediatrics, Center for Child and Adolescent Medicine, Heidelberg University Hospital, Heidelberg, Germany; 2 Screening Center, Bavarian Health and Food Safety Authority (LGL), Oberschleissheim, Germany; 3 Klinik Für Kinder- und Jugendmedizin, Helios Klinikum Erfurt, Erfurt, Germany; 4 Institute of Laboratory Medicine, Clinical Chemistry and Molecular Diagnostics, University Hospital, University of Leipzig, Leipzig, Germany; 5 Screening-Labor Hannover, Hannover, Germany; 6 Department of Clinical Chemistry, Hannover Medical School, Hannover, Germany; 7 Division of Laboratory Medicine, Centre for Children and Adolescents, Kinder- und Jugendkrankenhaus Auf der Bult, Hannover, Germany

**Keywords:** MCAD, Fatal disease course, Sudden neonatal death, Mortality, Neonatal screening

## Abstract

Medium-chain acyl-coenzyme A dehydrogenase (MCAD) deficiency is the most common disorder of mitochondrial β-oxidation of fatty acids resulting in hypoketotic hypoglycemia, hepatopathy, and often fatal outcome in undiagnosed children. Introduction of tandem mass spectrometry–based newborn screening programs in the late 1990s has significantly reduced morbidity and mortality in MCAD deficiency; however, neonatal death in individuals with early disease manifestation and severe hypoglycemia may still occur. We describe the fatal disease course in eight newborns with MCAD deficiency, aiming to raise awareness for early clinical symptoms and the life-saving treatment, and promote systematic post-mortem protocols for biochemical and genetic testing, necessary for correct diagnosis and counselling of the family if unexpected death occurred in the neonatal period.

*Conclusion*: Early newborn screening and awareness for clinical symptoms is lifesaving in MCAD deficiency, which may present with fatal neonatal crisis. Systematic post-mortem diagnostic protocols are needed for sudden neonatal deaths.

**What is Known:**

*• Medium-chain acyl-coenzyme A dehydrogenase (MCAD) deficiency identified by newborn screening has an excellent outcome.*

*• Fatal neonatal crises occur in the first days prior to screening.*

**What is New:**

*• Poor feeding, no monitoring of blood glucose, and homozygosity of the common gene variant (c.985A > G) are major risk factors for fatal neonatal crisis in MCAD deficiency.*

*• Post-mortem diagnostic protocols are indispensable for correct diagnosis and counselling of the family if unexpected death occurred in the neonatal period.*

## Introduction

Medium-chain acyl-coenzyme A (CoA) dehydrogenase (MCAD) catalyzes the initial step in the β-oxidation of medium-chain acyl-CoAs. MCAD deficiency results in a lack of energy production by reduced ketogenesis and gluconeogenesis and causes an accumulation of medium-chain CoAs and their corresponding acylcarnitines resulting in a characteristic biochemical pattern which is used for newborn screening (NBS) and selective metabolic diagnostics. MCAD is encoded by the *ACADM* gene and 201 pathogenic or likely pathogenic variants have been described with an additional 127 variants of uncertain significance (https://www.ncbi.nlm.nih.gov/clinvar/?term=Acadm%5Bgene%5D, accessed December 10, 2021).

In natural history, individuals with MCAD deficiency (OMIN #201,450) appear completely normal until the first metabolic decompensation which typically occurs within the first few months or years of life [1, 2] in catabolic episodes induced by prolonged fasting, recurrent vomiting, and/or intercurrent infectious disease. These episodes can precipitate life-threatening hypoglycemia, lethargy, reduced consciousness, seizures, and acute hepatopathy [1, 2]. Since implementation of tandem mass spectrometry in the 1990s, MCAD deficiency is part of most NBS programs worldwide [3] and thus is identified in the early postnatal period. Preventive measures, such as avoidance of fasting and use of emergency plans to provide carbohydrates orally or intravenously during catabolism, give these patients a favorable long-term outcome [4–6]. However, neonatal metabolic decompensation due to MCAD deficiency has been reported in the first hours and days of life before NBS results are available or even prior to sampling [6–9]. These episodes led to death in up to 5% of affected patients [6, 9] and potentially severe residual morbidity in survivors [10]. The present cohort study of neonatally deceased infants with MCAD deficiency since start of the unified expanded German NBS program in 2005 evaluated common elements in the clinical course, identified potential pitfalls, and recommends additional preventive measures which hopefully will further reduce neonatal mortality in this metabolic disorder.

## Patients and methods

To identify neonatally deceased patients with MCAD deficiency in Germany since start of the expanded national NBS program in 2005 until the present year (2021), German NBS laboratories (Hannover, Leipzig, München, and Heidelberg) and the German Society for Neonatal Screening (DGNS), collecting nationwide NBS cases communicated by the German NBS laboratories, were asked in April 2021 to report all recorded cases of neonatal death of patients with MCAD deficiency since 2005.

Data collection was performed anonymized using the data set of the multicenter observational NBS study in Southwest Germany (DRKS-ID: DRKS00013329; [4] ). The study was approved by the local ethics committee of the coordinating site (University Hospital Heidelberg, application no. S-104/2005). Following the study protocol, anonymized data of deceased patients are collected without informed consent of the caregivers to avoid re-traumatization of the families. The data set included NBS parameters, results of confirmatory diagnostics, and medical history (pregnancy, clinical data). The surviving sibling (patient 2b, Table 1) fulfilled all inclusion criteria [4] and parents gave informed written consent.

**Table 1:** Clinical characteristics

Child	Week of gestation	Birth weight (g)	APGAR	Information on feeding	Age at death (days after birth)	Medical history
1	40	3430	Missing	Poor feeding	4	Sudden unexpected death at home
2a	41	3320	10/10	Poor feeding	3	Sleepy at day 2, sudden unexpected death at maternity hospital, resuscitation without success
2b	41	3920	10/10	Normal	Survivor	n/a
3	41	3550	10/10	Poor feeding	3	Day 2: Lethargy, weight loss of 10%, blood glucose 2.1 mmol/l. Day 3 (64 h, maternity hospital): acute emergency, pulselessness, apnea, blood glucose not detectable (0 mmol/l), pH 6.73, base excess − 28 mmol/l, elevated liver enzymes. Unsuccessful resuscitation
4	Missing	3440	Missing	No information	6	At maternity/children’s hospital: multi-organ failure, lactic acidosis, blood glucose 0.7 mmol/l, suspected diagnosis: neonatal sepsis
5	39	2520	Missing	No information	3	Sudden unexpected death at home
6	40	3820	Missing	No information	3	No information available
7	39	3326	9/10	Poor feeding	6	Poor feeding, dry diapers, deterioration at home at day 5. Pulseless. Resuscitation by emergency team and in PICU, blood glucose 0.1 mmol/l. Death in multi-organ failure
8	39	3460	Missing	Normal	5	Sudden unexpected death at home

### Statistical analyses

Boschloo’s exact test was used to analyze two-way contingency tables, and Bonferroni alpha error adjustment was applied to correct *p*-values in multiple comparisons.

## Results

Since 2005, nine individuals with MCAD deficiency, one of them screened in another European country, have been reported by German NBS laboratories and DGNS. Median (range) age of death was 3.5 (3–6) days. Evaluation of the circumstances of death and comorbidities indicated that MCAD deficiency was causal in all but one patient (39 weeks of gestation, birth weight: 3430 g) who died at day 4 due to a total anomalous pulmonary venous connection. This patient was excluded from further analyses.

### Clinical data and medical history

Clinical data and medical history are summarized in Table 1. Infants were born at term with a median (range) of 40 (39–41) weeks of gestation, median (range) birth weight of 3435 (2520–3820) g, and unremarkable postnatal adaptation (Table 1). For five patients, information on feeding were reported (*n* = 3 missing): In four patients (80%), poor feeding preceded fatal decompensation. Two of them had been already discharged, while in the other two metabolic decompensation was recognized and/or sudden unexpected death occurred in the maternity hospitals. Severe hypoglycemia was found in all infants in whom blood glucose had been measured during crisis (Table 1).

### NBS process, results, and confirmatory diagnostics

In all but one patient (*n* = 7), NBS samples were taken prior to death at a median (range) age of 46 (38–72) hours, all adhering to the German regulatory specification (36–72 h; [11]; Table 2), and NBS results were available at median (range) of 5 (4–7) days. In patient #2a, no NBS sample was taken before death at the age of day 3. In the younger sibling, born 3 years later (#2b), MCAD deficiency was identified by NBS and subsequently confirmed. Retrospectively, MCAD deficiency was highly suggestive to have caused the older sibling’s (2a) sudden neonatal death, which had not been followed by any other diagnostics at that time.

**Table 2:** Results of NBS and genetic confirmation

Child	Age at NBS sampling (h)	Sample arrival NBS lab (days after birth)	NBS report (days after birth)	C8 (µmol/l) N** < 0.15		C10 (µmol/l) N** < 0.38	C10:1 (µmol/l) N** < 0.09	C6 (µmol/l) N** < 0.12	C0 (µmol/l) N** 4.3–57	C8/C2 N** < 0.01	C8/C10 N** < 1.39	C8/C12 N** < 1.9	Genetic testing
1	72	5	9	41.9		n/a	n/a	n/a	n/a	n/a	n/a	n/a	Homozygous c.985A > G
2a	Death before NBS samples was taken												Not done
2b	40	3	3	26.4		2.17	0.9	4.56	Missing	0.78	12.1	94	Homozygous c.985A > G
3	41	4	4	24.3		1.76	0.48	2.59	13	1.52	13.8	90	Homozygous c.985A > G
4	72	6	7	19.8		3.33	1.46	3.57	24	Missing	47.8	Missing	Homozygous c.985A > G
5	41	3	4	8.1		0.98	0.25	1.12	42	Missing	8.3	Missing	Homozygous c.985A > G
6	38	5	5	37.6		3.30	1.6	4.20	27	Missing	11.4	31	Homozygous c.985A > G
7	71	4	5	17		1.18	0.2	1.26	14	0.82	14.5	58	Homozygous c.985A > G
8	46	5	5	28.2		2.27	0.43	2.16	Missing	0.85	12.4	74	Missing
All*	*N* Median (range)												
	*N* = 7 46 (38–72)	*N* = 7 5 (3–6)	*N* = 7 5 (4–9)		*N* = 7 24.3 (8.1–41.9)	*N* = 6 2.02 (0.98–3.33)	*N* = 6 0.46 (0.2–1.6)	*N* = 6 2.38 (1.12–4.20)	*N* = 5 24 (13–42)	*N* = 3 0.85 (0.82–1.52)	*N* = 6 13.1 (8.3–47.8)	*N* = 4 66 (31–90)	*N* = 6 Homozygous c.985A > G

^*^Without individual #2b

^**^Normal values, Heidelberg NBS laboratory

All deceased newborns had massively elevated concentration of octanoylcarnitine (C8) in NBS (n = 7; median 24.3 µmol/l; reference < 0.15 µmol/l). Additionally, other medium-chain acylcarnitines and their ratios (Table 2) were also clearly elevated. Confirmatory genetic analysis revealed homozygosity for the classic pathogenic *ACADM* variant c.985A > G (Lys329Glu; Table 2) for all tested patients. 

### MCAD deficiency, birth prevalence, and neonatal mortality rate

According to published data from the German NBS program (annual reports of the DGNS [12] ) 9,941,846 newborns were screened in Germany from 2005 to 2018. In 968 of them, MCAD deficiency was confirmed after positive NBS [12]. This revealed a birth prevalence of about 1 in 10,300 newborns, in line with previous reports [4], and an overall neonatal MCAD mortality rate of 0.6% (Table 3). This is lower than that reported in an Australian unscreened population (7.5% [6]; *p* = 0.0065; Boschloo’s test; Table 3), but comparable to their screened cohort (2.4%; [6]; *p* = 0.42). Considering the percentage (47% [13], i.e. 455 of 968) of homozygosity for c.985A > G in screened individuals with MCAD deficiency in Germany, neonatal mortality rate for this genetic constellation was 1.3%, still in trend lower than in the unscreened Australian cohort ( [6]; *p* = 0.051; Boschloo’s test; Table 3).

**Table 3:** Neonatal mortality in MCAD deficiency. The Australian screened and unscreened cohort 1994–2004 [6] are compared to the German cohort 2005–2018 [12]

	Australia 1994–2004 Unscreened [6]	Australia 1994–2004 Screened [6]	Germany 2005–2018 Screened [12]	
Birth cohort (*n*)	1,685,000	810,000	9,941,846	
MCAD deficiency (*n*)	40	41	968 (all)	~ 455 (C.985A > G homozygous*
Reported neonatal death in individuals with MCAD deficiency (mortality rate)	3 (7.5%)	1 (2.4%)	6 (0.6%)	6 (1.3%)

^*^Estimated number of c.985A > G homozygous patients according to [13]

## Discussion

In individuals with MCAD deficiency, detection by NBS and subsequent specific measures such as avoidance of fasting and reliable supply of carbohydrates during catabolic situations prevent metabolic decompensations [4] and ensure excellent long-term outcome [4–6]. However, fatal episodes of severe hyperglycemia triggered by postnatal catabolism can still occur in the first hours and days of life [6, 9].

We evaluated all reported cases of neonatal death in individuals with MCAD deficiency since introduction of MCAD deficiency in the German regular NBS panel in 2005. In all patients, lethal metabolic decompensation occurred before NBS results could be reported or even before NBS sample was taken. However, mortality rate for screened individuals with MCAD deficiency was still lower than in the pre-screening era [6, 9], demonstrating the effect of advances in neonatology and especially in screening and prevention of neonatal hypoglycemia in recent years combined with the effect of NBS in prevention of neonatal metabolic crises after abnormal results have been obtained [4].

All deceased MCAD-deficient infants were term-born and all but one were eutrophic. They were born in maternity hospitals and routine neonatal assessment was normal in all but one patient, who was excluded from our analysis because of severe congenital heart disease. The remaining newborns had no evidence of concomitant diseases so that MCAD deficiency was most likely the exclusive and preventable cause of death. However, none of these patients had received a post-mortem autopsy. Additional underlying conditions, which may have contributed to the lethal outcome, can therefore not completely be excluded.

Apart from MCAD deficiency, none of the deceased newborns was at risk for hypoglycemia requiring monitoring of blood glucose and early feeding, which might have prevented early metabolic decompensation and death. Nevertheless, poor feeding, enhancing postnatal catabolism, which may cause metabolic decompensation, was reported in 80% of the patients with reliable nutritional information. As a further complicating factor, 50% of the neonates were already discharged from maternity hospital when fatal metabolic crisis or sudden death occurred.

NBS showed a pattern characteristic for MCAD deficiency in all patients, comparable to previous reports [13]. Genetic testing revealed for all homozygosity for the common severe *ACADM* gene variant (c.985 A > G) found in about 80% of symptomatically diagnosed patients in the pre-screening era [14] and in about 32% to 59% of neonatally screened individuals with MCAD deficiency [5, 13]. Thus, homozygosity for c.985 A > G is a major risk factor for neonatal death in MCAD patients. Therefore, a post-mortem metabolic and genetic testing is of importance for counselling affected families and to reduce the risk for neonatal death in siblings. Patient 2b in our cohort is the younger sibling of 2a, in whom NBS and further diagnosis following the sudden unexpected death had not been performed.

To prevent early deaths in neonates with unidentified MCAD deficiency, the following aspects have to be considered:

### Timely sampling and processing of NBS

Time of NBS sampling differs between national NBS programs [15] and ranges from, e.g. the German recommendation for NBS sampling (age 36 and 72 h of life; [11] ) to > 120 h of life in the UK [16]. However, although median age at NBS sampling in Germany decreased from 72 to 50 h since 2003, NBS is usually not reported before a median age of 5 days because transport time increased [4]. As knowledge of the diagnosis would most likely have prevented the reported neonatal deaths [1] and mortality rates in confirmed MCAD patients up to 25% have been reported [1, 2], all parties involved in NBS are challenged to process NBS as recommended to enable the NBS report as early as possible: First, NBS should be taken as early as recommended in the national regulatory, e.g., in Germany already at 36 h of life. Second, the sample has to be shipped from maternity hospitals to the NBS laboratory on the day of sampling. Third, national mail transportation time should be assured to be completed within two working days. Fourth, analysis time at NBS laboratory should not exceed 24 h, as recommended by German national regulatory for NBS [11]. In case of outpatient births or discharge from maternity units before the regular NBS sampling time, an additional early sample at discharge should be taken and parents should be informed to care about a timely regular NBS by the pediatrician or midwife.

### Prevention and/or early treatment of neonatal hypoglycemia

MCAD deficiency is an inherited metabolic disease with safe and effective treatment, if identified early. Prevention and/or early treatment of neonatal hypoglycemia is of utmost importance to avoid neonatal metabolic decompensation. The success of hypoglycemia prevention in maternity and neonatal units is underlined by the finding that fatal decompensation occurred in newborns without additional risk for hypoglycemia (such as prematurity, small for gestational age (SGA), large for gestational age (LGA), or maternal diabetes). Most patients described in this report were born at term and had normal weight so that specific monitoring of blood glucose or early feeding did not seem necessary.

However, in this cohort, the majority of deceased MCAD patients showed poor feeding and lethargy as an early sign of decompensation. Patient 2a had feeding difficulties and was sleepy on day 2, but no additional feeding was given at the maternity hospital. Patient 3 was lethargic on day 2, had difficulties with breastfeeding, and lost about 10% of weight. Her blood glucose was 2.1 mmol/l, which was considered normal for a newborn less than 48 h of age. Obstetricians, midwifes, neonatologists, and all pediatricians should be aware that every neonate with poor feeding needs special monitoring. Apart from the most frequent neonatal differential diagnoses such as perinatally acquired infections and congenital heart defects, metabolic causes should be considered. Thus, blood glucose and blood gases should be measured in every newborn with feeding difficulties or lethargy. Early feeding and follow-up of blood glucose concentrations, if reduced, can be lifesaving for MCAD deficient patients before diagnosis. Nevertheless, screening for hypoglycemia in asymptomatic newborns is discussed controversial as it results in lower rates of exclusive breastfeeding [17].

### Post-mortem investigations

MCAD deficiency, as several other inherited metabolic diseases, may present as sudden neonatal or infant death [18]. Finding the correct diagnosis in case of neonatal fatal metabolic decompensation or sudden unexpected death is of utmost importance because otherwise genetic counselling of the parents, including a reliable risk assessment for additional children, is not possible. If a metabolic disorder is suspected or not excluded, but no specific results have been obtained at the time of death, we recommend to collect the samples summarized in Table 4 [19]. This will also allow to identify more cases, as patient #2a, that may have been missed and remained unsolved without adequate post-mortem investigation.

**Table 4:** Post-mortem investigations after neonatal fatal metabolic decompensation or sudden unexpected death adapted according to [19]

Sample	Procedure and storage	Analysis
EDTA whole blood 3–10 ml	- > DNA isolation No centrifugation, freeze, or store at room temperature	Genetic testing (single-gene, panel, or exome analysis)
Serum and/or plasma 2–10 ml	Centrifugate immediately and freeze (− 20 °C) in several fractions (500 µl)	Laboratory and metabolic investigations
Dried blood	Collect on filter paper card, dry without heat at room temperature, store at 4–8 °C	Acylcarnitines and amino acids by tandem mass spectrometry Further metabolic and genetic testing
Urine 2–20 ml	Freeze (− 20 °C) immediately in several fractions (2 ml)	Laboratory and metabolic investigations
Culture fibroblasts	Skin biopsy, obtained up to 24 h post-mortem; may be stored 1–2 days in culture medium or NaCl 0.9%. Do not freeze	Genetic and enzymatic tests
Consider cerebrospinal fluid (CSF)	Freeze (ideally − 80 °C) immediately in several fractions (500 µl)	Laboratory and metabolic investigations

## Conclusion

In conclusion, MCAD deficiency, a treatable inherited metabolic disease, has been part of NBS programs worldwide for more than 20 years. Preventive measures allow an excellent outcome for screened individuals with MCAD deficiency. Early fatal neonatal metabolic decompensations occurring in the first days of life cannot be prevented by NBS. To further reduce mortality, we recommend sampling of NBS very early in the time frame recommended by national specifications, and additional NBS sampling for outpatient birth or early discharged children. Also, algorithms to prevent, diagnose, and treat hypoglycemia in neonates in maternity and neonatal units should be optimized. If unexpected neonatal death occurs despite these measures, there should be a specific post-mortem diagnostic protocol, which at least includes a dried blood spot card for NBS.

## Data Availability

Not applicable.

## Abbreviations

C8: Octanoylcarnitine
CoA: Coenzyme A
DGNS: German Society for Neonatal Screening
MCAD: Medium-chain acyl-coenzyme A dehydrogenase
NBS: Newborn screening
